# A strain defined as a novel species in the *Acinetobacter* genus co-harboring chromosomal associated *tet*(X3) and plasmid associated *bla*_NDM-1_ from a beef cattle farm in Hebei, China

**DOI:** 10.3389/fcimb.2025.1594982

**Published:** 2025-07-02

**Authors:** Qing Wang, Yanming Wei, Muhammad Shoaib, Yanhua Qiu, Chao Zhang, Guonian Dai, Honglin Lin, Weiwei Wang, Jiyu Zhang

**Affiliations:** ^1^ Lanzhou Institute of Husbandry and Pharmaceutical Sciences, Chinese Academy of Agricultural Sciences, Lanzhou, Gansu, China; ^2^ College of Veterinary Medicine, Gansu Agricultural University, Lanzhou, Gansu, China; ^3^ Key Laboratory of New Animal Drug Project of Gansu Province, Lanzhou, Gansu, China; ^4^ Key Laboratory of Veterinary Pharmaceutical Development, Ministry of Agriculture and Rural Affairs, Lanzhou, Gansu, China

**Keywords:** antibiotic resistance genes (ARGs), *tet*(X), *bla*_NDM_, *Acinetobacter*, multi-drug resistance, p*dif* module

## Abstract

**Introduction:**

The co-existence phenomenon of antibiotic resistance genes (ARGs), particularly of last-resort antibiotics in multi-drug resistant (MDR) bacteria, is of particular concern in the least studied bacterial species.

**Methods:**

In 2023, strain M2 was isolated from the sludge sample at a commercial bovine farm in Hebei province, China, using a MacConkey plate containing meropenem. PCR amplification and Sanger sequencing verified it co-carrying *bla*_NDM_ and *tet*(X) genes. It was classified within the Acinetobacter genus by MALDI-TOF-MS and 16S rDNA analyses. Whole-genome sequencing (WGS) was performed on the Oxford Nanopore platform, with species-level identification via ANI and dDDH. Antimicrobial susceptibility testing was performed against 20 antibiotics. Conjugation assays employed the filter-mating method using *E. coli* J53 and *Salmonella* LGJ2 as recipients.

**Results:**

This strain was confirmed as a novel species of *Acinetobacter* genus, showing resistance to meropenem, ampicillin, ceftazidime, cefepime, gentamicin, kanamycin, fosfomycin, imipenem, ertapenem, and tetracycline. Despite carrying *tet*(X3), it remained susceptible to tigecycline, omadacycline, and doxycycline. The genome carried 11 ARG types, multiple metal resistance genes (MRGs), and virulence factor (VF) genes. The *bla*_NDM-1_ was located in a skeleton, IS*Aba125-bla*_NDM-1_-*ble*_MBL_-*trpF*, which was carried by an IS*Aba*14-mediated rolling-circle-like structure in pM2-2-NDM-1 (rep_cluster_481). Integrative and conjugative element (ICE) and multiple pd*if* modules (driven by the XerCD site-specific recombination (XerCD SSR) system), which were associated with the mobilization of resistance determinants, were identified in this plasmid. Chromosomal *tet*(X3) was mediated by IS*Vsa3*, forming a skeleton, IS*Vsa3-XerD-tet* (X3)*-res-ISVsa3*.

**Discussion:**

The co-occurrence of *bla*_NDM_ and *tet*(X) in a novel species of the *Acinetobacter* genus hints that substantial undiscovered bacteria co-carrying high-risk ARGs are concealing in the agroecological system, which should cause particular concern.

## Introduction

*Acinetobacter*, an organism known for its natural resistance and remarkable ability to acquire additional resistance factors, is clinically significant (Peleg et al., 2008). It can survive on the surfaces of medical devices or host tissues in hospital environments via biofilms (Dijkshoorn et al., 2007; Shi et al., 2024). Its spread among patients can occur through the hands of healthcare personnel and the cross-contamination of medical devices, making it a significant contributor to nosocomial infections (Ching et al., 2024; Shi et al., 2024). In recent years, there has been a significant increase in reports of *Acinetobacter* genus from medical or agricultural systems carrying ARGs, even the genes conferring resistance to last-resort antibiotics (Poirel and Nordmann, 2006; He et al., 2019; Cheng et al., 2021; Cheng et al., 2023). Notably, after 2018, reports regarding the co-occurrence of the genes conferring resistance to last-resort antibiotics in *Acinetobacter* (Liu et al., 2021; Tang et al., 2021; Cheng et al., 2023; Opazo-Capurro et al., 2024; Long et al., 2025; Mallonga et al., 2025), *Klebsiella* (Seiffert et al., 2014), and *Escherichia* (Delgado-Blas et al., 2016; Lu et al., 2022) genera have become more frequent. Studies have revealed that the co-occurrence phenomenon in the *Acinetobacter* genus primarily involves *bla*_OXA-58_, *tet*(X3), *te*t(X5), *te*t(X6), *tet*(X7), *bla*_NDM-1_, *bla*_NDM-3_ and *bla*_NDM-5_ genes (Tang et al., 2021; Gutiérrez et al., 2024; Opazo-Capurro et al., 2024; Jia et al., 2025; Long et al., 2025; Mallonga et al., 2025; Mmatli et al., 2025). Among these genes, *bla*_NDM_ and *tet*(X) are the most prevalent in clinical and agricultural settings as a co-existence unit. This co-existence was mainly found in *A. baumannii*, *A. indicus*, *A. towneri*, and *A. bereziniae* (Opazo-Capurro et al., 2024; Jia et al., 2025; Long et al., 2025; Mallonga et al., 2025), with *A. baumannii* being the dominant host. Notably, the increasing species in the *Acinetobacter* genus co-carry the genes conferring resistance to last-resort antibiotics, particularly scarce species. In addition, current research suggests that the co-occurrence is predominantly found in Asia, primarily within meat production systems such as poultry farms, pig farms, and slaughterhouses (Tang et al., 2021; Long et al., 2025), with a secondary presence in healthcare systems (Mallonga et al., 2025).

A prominent manifestation of antimicrobial resistance (AMR) involves the global distribution of carbapenem-resistant bacteria (CRB), such as the *bla*_NDM_ gene. This gene encodes New Delhi Metallo-β-lactamase (NDM) with the capability of hydrolyzing nearly all β-lactam antibiotics, including carbapenems, the last-resort therapeutic agents for multidrug-resistant bacterial infections. The *bla*_NDM_ gene has been detected in at least 11 bacterial genera. Although *bla*_NDM_ has been observed in the bacterial chromosome, it predominantly resides on plasmids (Toleman and Walsh, 2012). This gene has been associated with over 20 distinct plasmid types, including major types such as IncFIB, IncFII, IncA/C (IncC), IncX3, IncH, and IncL/M, as well as untyped plasmids (Wang et al., 2017; Acman et al., 2022; Kikuchi et al., 2022). The *tet*(X) can degrade all tetracyclines, particularly tigecycline, one of the last options for treating carbapenem-resistant bacteria (CRB). Similarly, the *tet*(X) exhibits the distribution characteristic of cross-bacteria genera and cross-plasmid types (He et al., 2019; Sun et al., 2019). Its co-existence with *bla*_NDM_ compromises the efficacy of last-resort antibiotics, posing a significant challenge to antimicrobial stewardship and infection control strategies.

Here, we exhibit *Acinetobacter* sp. M2 co-carrying *bla*_NDM-1_ and *tet*(X3). Furthermore, the ICEs and p*dif*-ARG modules related to the horizontal transfer of ARGs have been identified in the pM2-2-NDM-1 of this strain. ICE is an important member of the bacterial mobile genetic elements (MGEs), which is integrative to the bacterial chromosome and encodes fully functional conjugation machinery and is thus self-transmissible between bacterial cells (Bi et al., 2012; Li et al., 2018; Lin et al., 2020; Wang et al., 2024). The p*dif*-ARG module is flanked by XerCD site-specific recombination sites (Lin et al., 2020; Shao et al., 2023). These modules have been found in plasmids of multiple bacterial genera, regarded as MGEs driven by the XerCD SSR to facilitate horizontal gene transfer (Lin et al., 2020; Shao et al., 2023). XerC and XerD are encoded by numerous bacteria, usually in pairs, and are homologous recombinases (tyrosine recombinase family) that catalyze the cleavage of two consecutive pairs of DNA strands and exchange with a restriction site, *dif*, located in the terminus region of the chromosome (Mindlin et al., 2019; Lin et al., 2020; Shao et al., 2023). Typically, the *dif* site is a 28 bp site consisting of two inverted repeat 11 bp Xer binding motifs (the left and the right regions of C/D and D/C) separated by a six bp interval called the central region (Mindlin et al., 2019; Lin et al., 2020; Shao et al., 2023). A monomer of XerC and XerD each binds to an 11 bp semi-binding site (Mindlin et al., 2019; Lin et al., 2020; Shao et al., 2023). The *dif* sites in plasmids are called p*dif* sites and appear multiple times in a plasmid (Shao et al., 2023).

## Results

### Source

In August 2023, we isolated a meropenem-unsusceptible strain M2 from a sludge sample at a commercial cattle farm in Hebei, China.

### The identification of bacterial species

PCR amplification and Sanger sequencing verified that this strain co-carried *bla*_NDM_ and *tet*(X) genes. This strain was classified as *Acinetobacter* genus using MALDI-TOF-MS and 16S rDNA. Subsequently, this strain was sequenced using whole-genome sequencing (WGS) on the Oxford Nanopore platform (long-read sequencing technology). The analysis confirmed 99.03% completeness and 0.89% contamination in this genome assembly (Acinetobacter sp. A2 genome assembly ASM4853755v1 - NCBI - NLM). To confirm the species of this genome, the Average Nucleotide Identity (ANI) match was performed using the NCBI annotation service. The result found that no genome showed >95% ANI with this genome (Acinetobacter sp. A2 genome assembly ASM4853755v1 - NCBI - NLM). The search on the DSMZ (Deutsche Sammlung von Mikroorganismen und Zellkulture) platform using the type strain genome server (TYGS) identified this genome as a potential new species, with the best match *A. seohaensis* DSM 16313 (34.1% dDDH), *A. towneri* DSM 14962 (33%), and *A. indicus* CIP 110367 (24%). This genome best matched *A. towneri* DSM 14962 = CIP 107472 (0.98559 Z-Score, 87.32% ANI), *A. towneri* DSM 14962 = CIP 107472 DSM 14962(0.98498, 87.27%), and *A. tibetensis* Y-23 (0.94622, 77.69%) on the JSpeciesWS platform using the tetra correlation search (TCS). Subsequently, we employed Mash (a k-mer-based rapid sequence alignment tool) on the Pathogenwatch platform. The analysis revealed that this genome best matched NZ_JAAZQX010000010.1, with a mash distance of 0.0147665, a p-value of 0, and matching hashes of 579/1000. We found that the NZ_JAAZQX010000010.1 was one of the contigs in the genome, GCA_012371325.1 (this is the only genome identified as *Acinetobacter* sp. *A2* in the NCBI genome database). Thus, the ANI calculation on the EZBioCloud platform was executed for GCA_012371325.1 and genome M2. We found that these genomes exhibited 98.35% ANI. Furthermore, the digital DNA-DNA hybridization (dDDH) calculation of these genomes was performed on the DSMZ platform using the genome-to-genome distance calculator (GGDC). The analysis revealed that these genomes exhibited 85.5% dDDH. The thresholds of ANI and dDDH are usually applied to define genomic species (95–96 and 70 % for ANI and dDDH, respectively) (Meier-Kolthoff et al., 2013). However, 98.35% ANI and 85.5% dDDH are beyond defined thresholds, suggesting genome M2 and GCA_012371325.1 are very close but may belong to different species (Riesco and Trujillo, 2024). Furthermore, *Acinetobacter* sp. *A2* belongs to an unclassified species of the *Acinetobacter* genus in the family *Moraxellaceae* (NCBI taxonomy database). Thus, it is classified only at the genus level, not at the species level. As a result, although strain M2 is closest related to *Acinetobacter* sp. *A2* (Taxonomy ID: 362457, Acinetobacter sp. A2 - NCBI - NLM), it should be defined as a novel species under the *Acinetobacter* genus.

### Antibiotic resistance phenotype

The antimicrobial susceptibility testing showed that the strain M2 was resistant to meropenem (16 mg/L), ampicillin (128 mg/L), ceftazidime (>1024 mg/L), cefepime (128 mg/L), gentamicin (128 mg/L), kanamycin (512 mg/L), fosfomycin (512 mg/L), imipenem (16 mg/L), ertapenem (32 mg/L) and tetracycline (16 mg/L) (**Table 1**). However, it was susceptible to aztreonam, chloramphenicol, colistin, ciprofloxacin, sulfamethoxazole, azithromycin, doxycycline, tigecycline, amikacin and omadacycline (**Table 1**).

**Table 1:** MIC and ARGs of the strain M2.

Antibiotics	MIC (mg/L)	Interpretation	ARGs
Meropenem (MEM)	16	resistant	*bla*_NDM-1_
Aztreonam (ATM)	<2	susceptible	N/O
Ampicillin (AMP)	128	resistant	*bla*_NDM-1_
Ceftazidime (CAZ)	>1024	resistant	*bla*_NDM-1_
Cefepime (FEP)	128	resistant	*bla*_NDM-1_
Gentamicin (GEN)	128	resistant	*aac(3)-IId*
Chloramphenicol (CHL)	<2	susceptible	N/O
Colistin (CL)	<2	susceptible	N/O
Kanamycin (KAN)	512	resistant	*aph(3’)-Ia*
Fosfomycin (FOS)	512	resistant	N/O
Ciprofloxacin (CIP)	<2	susceptible	N/O
Sulfamethoxazole (SXT)	64	susceptible	*sul2*
Azithromycin (AZM)	<2	susceptible	*msr*(E)
Tetracycline (TET)	16	resistant	*tet*(X3), *tet*(39)
Doxycycline (DOX)	8	susceptible	*tet*(X3), *tet*(39)
Tigecycline (TGC)	<2	susceptible	*tet*(X3)
Imipenem (IPM)	16	resistant	*bla*_NDM-1_
ertapenem (ETP)	32	resistant	*bla*_NDM-1_
Amikacin (AN)	8	susceptible	*aph(3’)-VI*
Omadacycline (OMC)	8	susceptible	*tet*(X3)
antibiotics not included in this experiment	–	–	*aph(6)-Id*, *aph(3’’)-Ib*, *mph*(E)

N/O, Not observed known resistance genes in this study.

### The horizontal transferability of plasmid

The previous report regarding successful conjugation in the *Acinetobacter* plasmid remains rare. However, successful conjugation in the *A. indicus* plasmid was observed in our laboratory. In this study, we used the same parameters and tried new ones. However, the horizontal transfer of the plasmid carrying *bla*_NDM_ was not observed.

### Genetic diversity analysis

**Figure 1:**
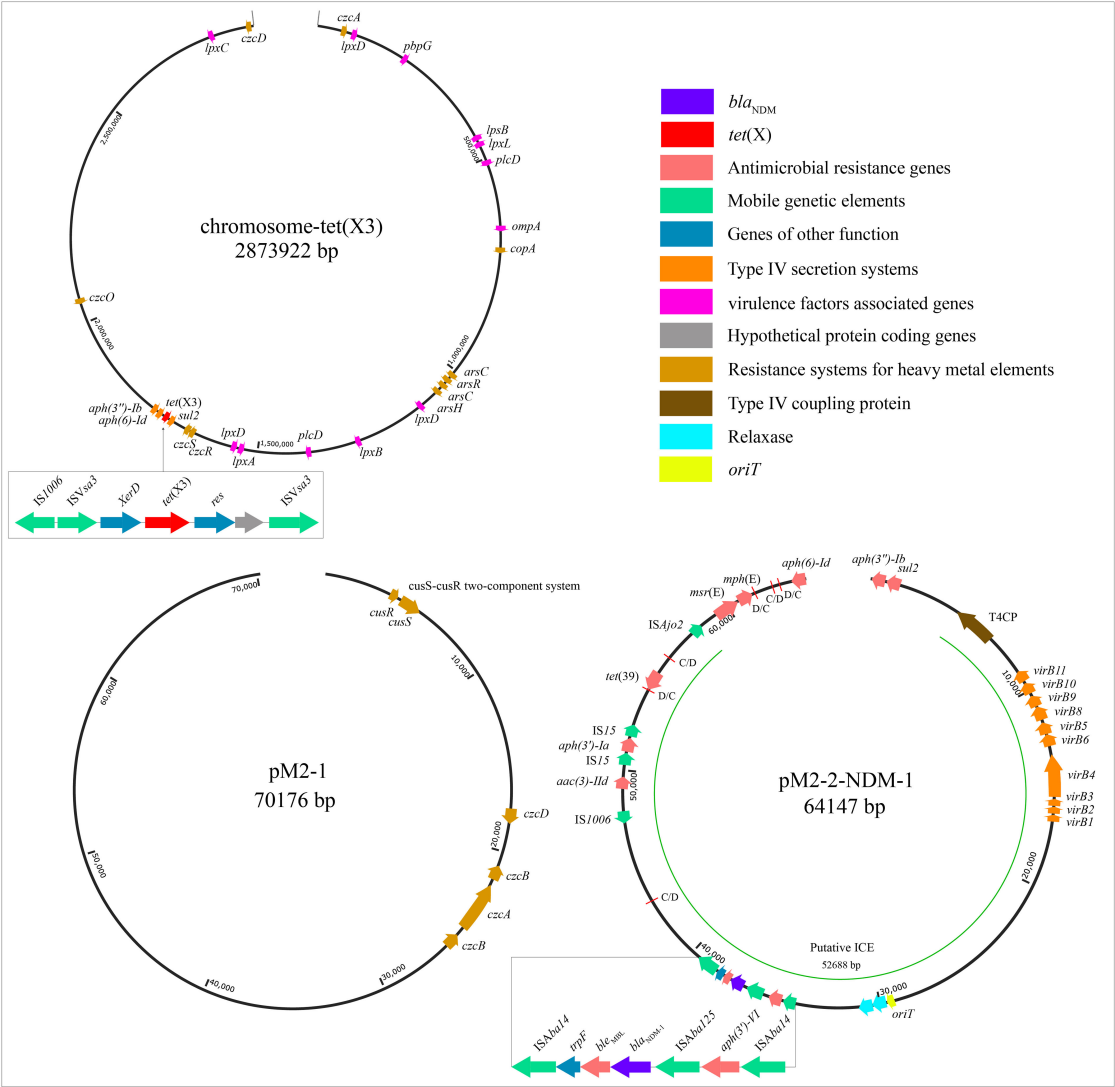
Structural features of the elements in the chromosome and plasmids. This figure shows the backgrounds of *bla*_NDM_ and *tet*(X), ARGs, MRGs, genes encoding VFs, putative ICE region (*oriT*, T4SS, T4CP, relaxase), and p*dif* modules. In pM2-2-NDM-1, the red lines mark the C/D and D/C of the p*dif* modules.

The WGS revealed that strain M2 contained one circular chromosome genome (2873922 bp, 41.5% GC content) and two circular plasmid genomes (pM2-1: 70176 bp, 40.2%; pM2-2-NDM-1: 64147 bp, 38.7%) (**Table 2**; **Figure 1**). The GC content of pM2-2-NDM-1 was lower than chromosome, hinting that they originated from different hosts. The GC content can serve as a marker to measure the origin of plasmids, indicating the potential horizontal transfer of plasmids (Tang et al., 2021; Long et al., 2025). The chromosome of the strain M2 carried multiple ARGs, including *tet*(X3), *aph(6)-Id, aph(3’’)-Ib, and sul2* (**Figure 1**). In addition to ARGs, this chromosome carried MRGs against arsenic, mercury, and copper, as well as genes encoding VFs related to bacterial adhesion, invasion, enzyme, immune evasion, and serum resistance (**Table 2**: **Figure 1**). The pM2–1 did not carry ARGs but harbored the genes related to copper resistance and the CusS-CusR two-component regulatory system (TCS) (**Table 2**: **Figure 1**). The pM2-2-NDM-1 carried various ARGs, such as *bla*_NDM-1_, *aac(3)-IId*, *aph(6)-Id*, *aph(3’)-Ia*, *aph(3’’)-Ib*, *aph(3’)-VI*, *msr*(E), *mph*(E), *sul2*, and *tet*(39) (**Table 2**: **Figure 1**).

**Table 2:** Genomic characteristics of the strain M2.

M2	Inc	Size (bp)	GC Content	ARGs (identity %)	MRGs	VFs
Chromosome-*tet*(X3)	–	2873922	41.5%	*aph(6)-Id* (100%), *aph(3’’)-Ib* (100%), *sul2* (100%), *tet*(X3) (100%)	Arsenic: *arsC*, *arsR*, *arsH*	adhesion and invasion: *ompA* Enzyme: *plcD* Immune evasion: *lpsA, lpxA, lpxB, lpxC, lpxD, lpxL*, Serum resistance: *pbpG*
					Mercury: *merR*	
					Copper: *copA*, *czcA*, *czcR*, *czcS*, *czcO*, *czcD*	
pM2-1	rep_cluster_1656	70176	40.2%	N/O	Copper: *czcD*, *czcB*, *czcD*, *cusR*, *cusS*	N/O
pM2-2-NDM-1	rep_cluster_481	64147	38.7%	*aac(3)-IId* (99.88%), *aph(6)-Id* (100%), *aph(3’)-Ia* (100%), *aph(3’’)-Ib* (100%), *aph(3’)-VI* (100%), *bla*_NDM-1_ (100%), *msr*(E) (100%), *mph*(E) (100%), *sul2* (100%), *tet*(39) (99.82%)	N/O	N/O

VFs, Virulence factors associated genes. N/O, Not observed known genes in this study. The identity value (%) was identified using ResFinder.

### Plasmid typing

The analysis of the pM2–1 and pM2-2-NDM-1 on the Pathogenwatch platform (homology-based alignment) revealed that they could not be identified as any known replicon type. Subsequently, the analysis of these plasmids on the Galaxy platform using MOB-typer revealed that pM2-2-NDM-1 was typed to rep_cluster_481 and predicted as conjugative, and pM2–1 was typed to rep_cluster_1656 and predicted as mobilizable. Furthermore, these plasmids were searched on the PLSDB database. The result revealed that pM2-2-NDM-1 exhibited the highest identity (>99.3%) with five plasmids. Among these, the sizes of three plasmids <5000 bp could not be identified as any known replicon type. The remaining two plasmids (NZ_CP051870.1 and NZ_CP051876.1) with the same size of 48239 bp were sourced from *A. baumannii*, typed to rep_cluster_481 and predicted as conjugative. However, these plasmids did not carry *bla*_NDM_ or *tet*(X). In addition, the search of pM2–1 found that no plasmid exhibited identity.

### Mobile genetic elements

**Figure 2:**
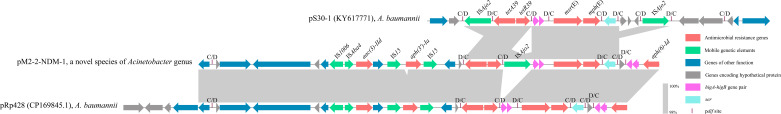
Comparison analysis of the p*dif* module island. The C/D and D/C sites of each p*dif* module are shown in **Table 3**.

The analysis of the pM2-2-NDM-1 on the ICEfinder platform identified a putative ICE region of 52688 bp (4758–57445 bp) containing the origin site of DNA transfer (*oriT*, 29162–29538 bp), T4SS (*virB11*, *virB10*, *virB9*, *virB8*, *virB5*, *virB6*, *virB4*, *virB3*, *virB2*, and *virB1*), T4CP (Type IV Coupling Proteins), relaxase (*mobC* and MOBP), integrase, and ARGs (*bla*_NDM-1_, *aac(3)-IId*, *aph(3’)-Ia* and *tet*(39)) (**Figure 1**). Furthermore, six p*dif* sites were identified in the pM2-2-NDM-1, and they formed five p*dif* modules including three p*dif*-ARGs modules (p*dif*-*aac(3)-IId*-*aph(3’)-Ia*, p*dif*-*tet*(39) and p*dif*-*msr*(E)-*mph*(E)), p*dif*-ser (a serine recombinase) module and p*dif*-hp (hypothetical protein) module (**Figures 1**, **2**). These modules collectively build a p*dif* module island. This inevitably leads to sharing the internal C/D or D/C sites to form two types of p*dif* modules (Blackwell and Hall, 2017), one flanked by a C/D and a D/C site, and the other type flanked by a D/C and a C/D site. We searched the p*dif*-*aac(3)-IId*-*aph(3’)-Ia* module in the NCBI database and found that only one plasmid (pRp428) was highly similar (coverage 99% and identity 100%) to this module. The p*dif*-*tet*(39), p*dif*-*msr*(E)-*mph*(E) and p*dif*-ser modules were reported in pS30-1 (Blackwell and Hall, 2017). Thus, we used these plasmids and pM2-2-NDM-1 to construct the linear comparison (**Figure 2**) and identified p*dif* sites (**Table 3**). The p*dif*-*aac(3)-IId*-*aph(3’)-Ia*, p*dif*-*tet*(39), p*dif*-*msr*(E)-*mph*(E), p*dif*-ser and p*dif*-hp modules were identified in pRp428. Among these, the p*dif*-*tet*(39), p*dif*-ser, and p*dif*-hp modules were identical (100% coverage and identity) to those in pM2-2-NDM-1. The p*dif*-*aac(3)-IId*-*aph(3’)-Ia* module exhibited 98.64% identity to that in pM2-2-NDM-1. A gap of 178 bp existed in the non-coding region between left-hand IS*15* and *aph(3’)-Ia*. The p*dif*-*tet*(39) and p*dif*-ser modules in pS30–1 were identical (100% coverage and identity) to those in pM2-2-NDM-1. Interestingly, the p*dif*-*msr*(E)-*mph*(E) module in pRp428 and pS30–1 only carried *msr*(E)-*mph*(E) gene pair. This structure was commonly seen in previous reports (Blackwell and Hall, 2017; Shao et al., 2023). However, the p*dif*-*msr*(E)-*mph*(E) module in pM2-2-NDM-1 additionally carried IS*Ajo2* and *higA*-*higB*. The p*dif*-*higA*-*higB* and p*dif*-IS*Ajo2* modules were independently present in pS30-1. However, the C/D and D/C sites flanking *higA*-*higB* and IS*Ajo2* were not found in pM2-2-NDM-1. In addition, the D/C site is located upstream of *msr*(E) in the p*dif*-*msr*(E)-*mph*(E) modules of pRp428 and pS30-1. However, the C/D site is located upstream of IS*Ajo2* in the p*dif*-*msr*(E)-*mph*(E) module of pM2-2-NDM-1. The analysis of p*dif* sites (**Table 3**) found that the base variant appeared in the D/C sites of the p*dif*-ser module of pS30-1. The C/D and D/C sites of the remaining p*dif* modules of pRp428 and pS30–1 were identical to those in pM2-2-NDM-1.

**Table 3:** The C/D and D/C sites of p*dif* modules in **Figure 2**.

pdif-aac(3)-IId-aph(3’)-Ia	C/D			D/C		
	Left	Center	Right	Left	Center	Right
pM2-2-NDM-1	GCTTCGGATAA	GAGTTG	CTATTTTAAAT	ATTTCGTATAA	GGTGTA	TTATGTTAATT
pRp428	GCTTCGGATAA	GAGTTG	CTATTTTAAAT	ATTTCGTATAA	GGTGTA	TTATGTTAATT
p*dif*-*tet*(39)	C/D			D/C		
	Left	Center	Right	Left	Center	Right
pM2-2-NDM-1	ATTTCGTATAA	GGTGTA	TTATGTTAATT	ATTTAACATAA	TGGCTG	TTATGCGAAAC
pS30-1	ATTTCGTATAA	GGTGTA	TTATGTTAATT	ATTTAACATAA	TGGCTG	TTATGCGAAAC
pRp428	ATTTCGTATAA	GGTGTA	TTATGTTAATT	ATTTAACATAA	TGGCTG	TTATGCGAAAC
p*dif*-*msr*(E)*-mph*(E)	C/D			D/C		
	Left	Center	Right	Left	Center	Right
pM2-2-NDM-1	ATTTAACATAA	TGGCTG	TTATGCGAAAC	ATTTAACATAA	AATTTC	TTATGTGAAGT
pS30-1	ATTTAACATAA	TGGCTG	TTATGCGAAAC	ATTTAACATAA	AATTTC	TTATGTGAAGT
pRp428	ATTTAACATAA	TGGCTG	TTATGCGAAAC	ATTTAACATAA	AATTTC	TTATGTGAAGT
p*dif*-*ser*	C/D			D/C		
	Left	Center	Right	Left	Center	Right
pM2-2-NDM-1	ATTTAACATAA	AATTTC	TTATGTGAAGT	AATTCGTATAA	CGTGTA	TTATGTTAATT
pS30-1	ATTTAACATAA	AATTTC	TTATGTGAAGT	AGTTCGTATAA	TACGTA	TCATATTAATT
pRp428	ATTTAACATAA	AATTTC	TTATGTGAAGT	AATTCGTATAA	CGTGTA	TTATGTTAATT
p*dif*-hp	C/D			D/C		
	Left	Center	Right	Left	Center	Right
pM2-2-NDM-1	AATTCGTATAA	CGTGTA	TTATGTTAATT	ATTTAACATAA	TGGCGA	TTATACGAATC
pRp428	AATTCGTATAA	CGTGTA	TTATGTTAATT	ATTTAACATAA	TGGCGA	TTATACGAATC

The highlighted bases in red differ from the C/D and D/C sites of p*dif* modules in pM2-2-NDM-1.

### Plasmid analysis

**Figure 3:**
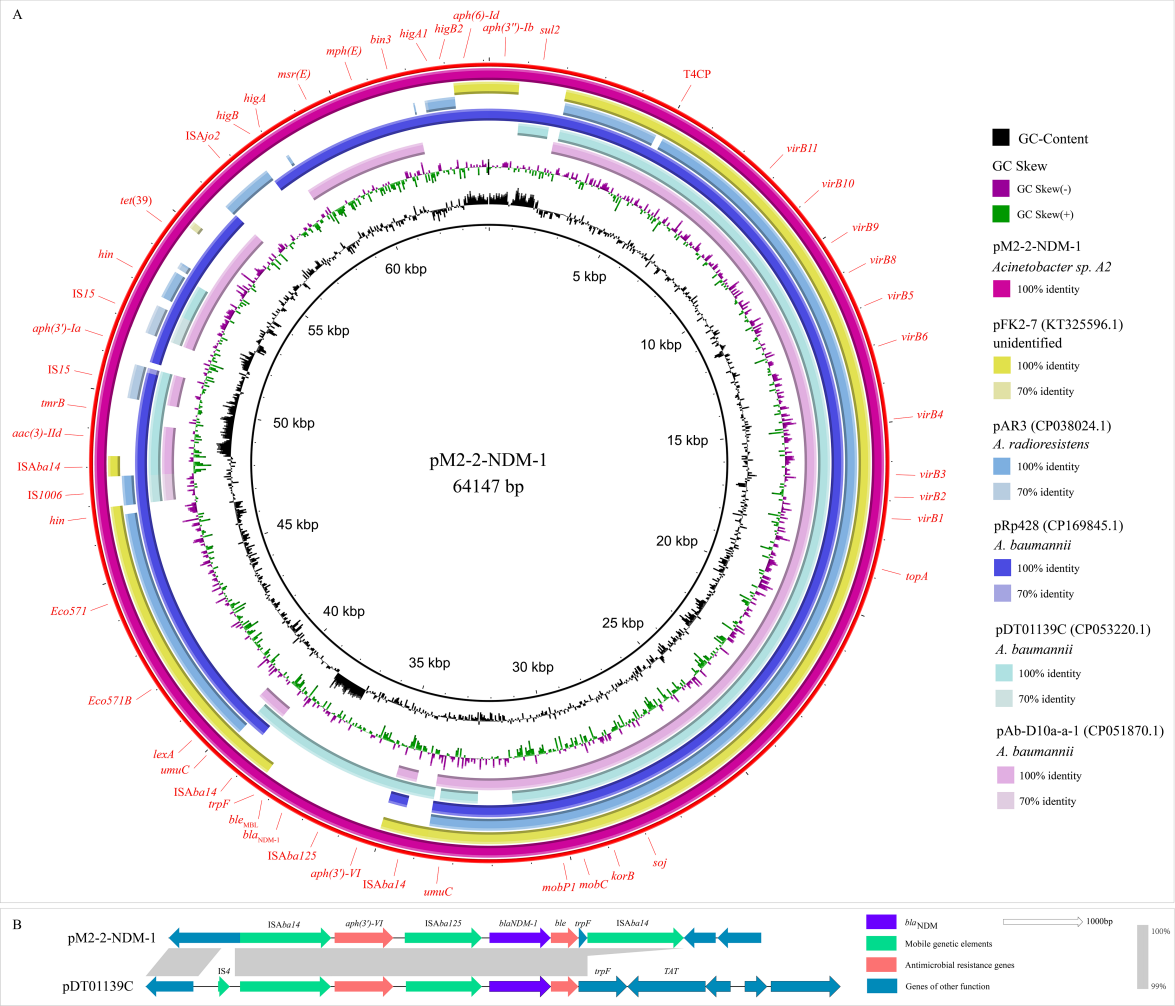
Plasmid analysis. **(A)**: Circular comparison between pM2-2-NDM-1 and the similar plasmids from the NCBI database. **(B)**: Comparison analysis of the *bla*_NDM-1_ skeletons from pM2-2-NDM-1 and pDT01139C.

BLASTn analysis in the GenBank database found that pM2-2-NDM-1 was similar to pRp428 (100% identity and 90% coverage), pAb-D10a-a (99.91% identity and 68% coverage), pDT01139C (99.07% identity and 67% coverage), pFk2-7 (98.5% identity and 68% coverage), and pAR3 (99.21% identity and 66% coverage) (**Figure 3A**). Among these, three plasmids (pRp428, pAb-D10a-a, and pDT01139C) were sourced from *A. baumannii*, pAR3 from *A. radioresistens*, and pFk2–7 from an unidentified strain. These plasmids and pM2-2-NDM-1 were used to build an alignment analysis (**Figure 3A**). The analysis found that the conserved region contained *oriT* and the genes encoding T4SS, T4CP, and relaxase. The non-conserved regions included ARGs, p*dif*-ARG modules, and IS elements. Only pDT01139C carried *bla*_NDM_ in the genomes from the public database. Thus, pM2-2-NDM-1 and pDT01139C genomes were used to build a comparison analysis of the *bla*_NDM_ skeleton (**Figure 3B**). The analysis found that the *trpF* in the *bla*_NDM-1_ framework of pDT01139C consisted of 636 bp. The *trpF* in the *bla*_NDM-1_ framework of pM2-2-NDM-1 consisted of 114 bp. Furthermore, no IS element could be identified downstream of the *bla*_NDM-1_ framework in pDT01139C.

### Phylogenetic analysis

**Figure 4:**
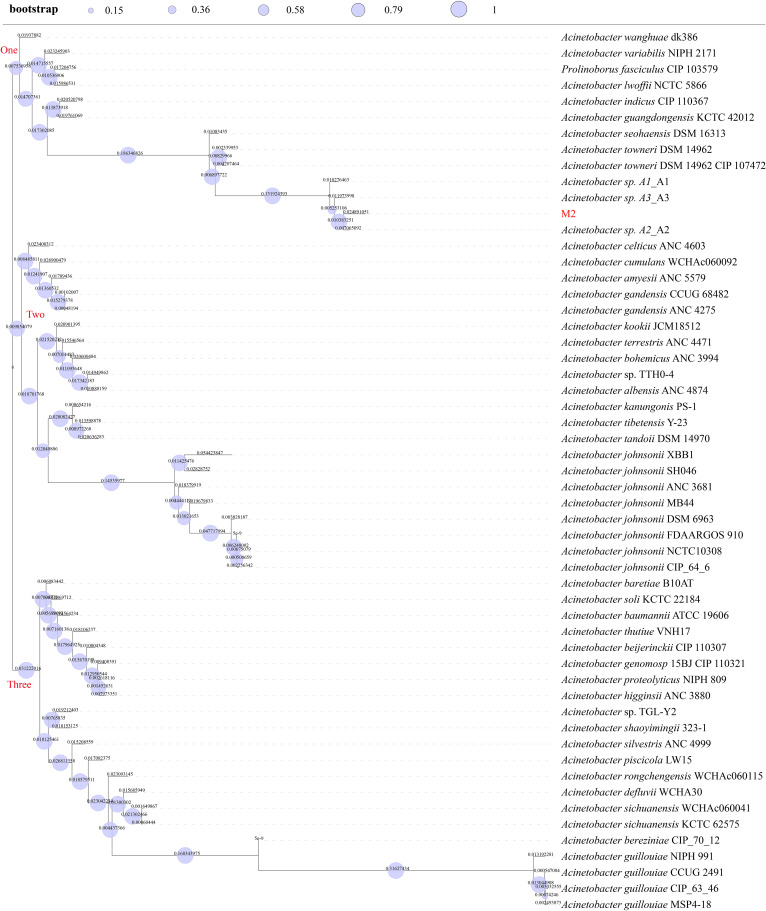
Phylogenetic tree. Bootstrap values were shown on the branch of this tree as circles. Branch lengths were displayed as numbers on each branch of this tree.

Three genomes were classified as *Acinetobacter* sp. *A1* (GCA_012371315.1)*, A2* (GCA_012371325.1)*, and A3* (GCA_012371415.1) in the NCBI genome database. A total of 51 genomes closely related to genome M2 were retrieved from the JSpesiesWS platform (TCS) and DSMZ platform (TYGS). Subsequently, the above genomes and genome M2 were used to build a phylogenetic tree (**Figure 4**). Phylogenetic analysis revealed that this tree was diverged into three branches, highlighting three different ancestors. In the branch one, *Acinetobacter* sp. *A1-A3* (A1-A3), *A. towneri* (DSM 14962 and DSM 14962 CIP 107472), and *A. seohaensis* (DSM 16313) were diverged from a clade, indicating their high homology. Among this, genomes M2 and A2 were diverged from a leaf node, suggesting they originated from a most recent common ancestor. Branch length quantifies accumulated genetic divergence from the most recent common ancestor. Genome M2 has higher genetic variability than A2 (branch length, 0.025 (M2) > 0.00707 (A2)), hinting that it undergoes prolonged independent evolution and accelerated evolution derived by selective pressure. Like the *Acinetobacter* sp. *A2*, the *Acinetobacter* sp. *A1* (Taxonomy ID: 401467) and *Acinetobacter* sp. *A3* (Taxonomy ID: 2725492) belong to unclassified species of the *Acinetobacter* genus in the family *Moraxellaceae*.

### The genetic context of *bla*_NDM-1_

**Figure 5:**
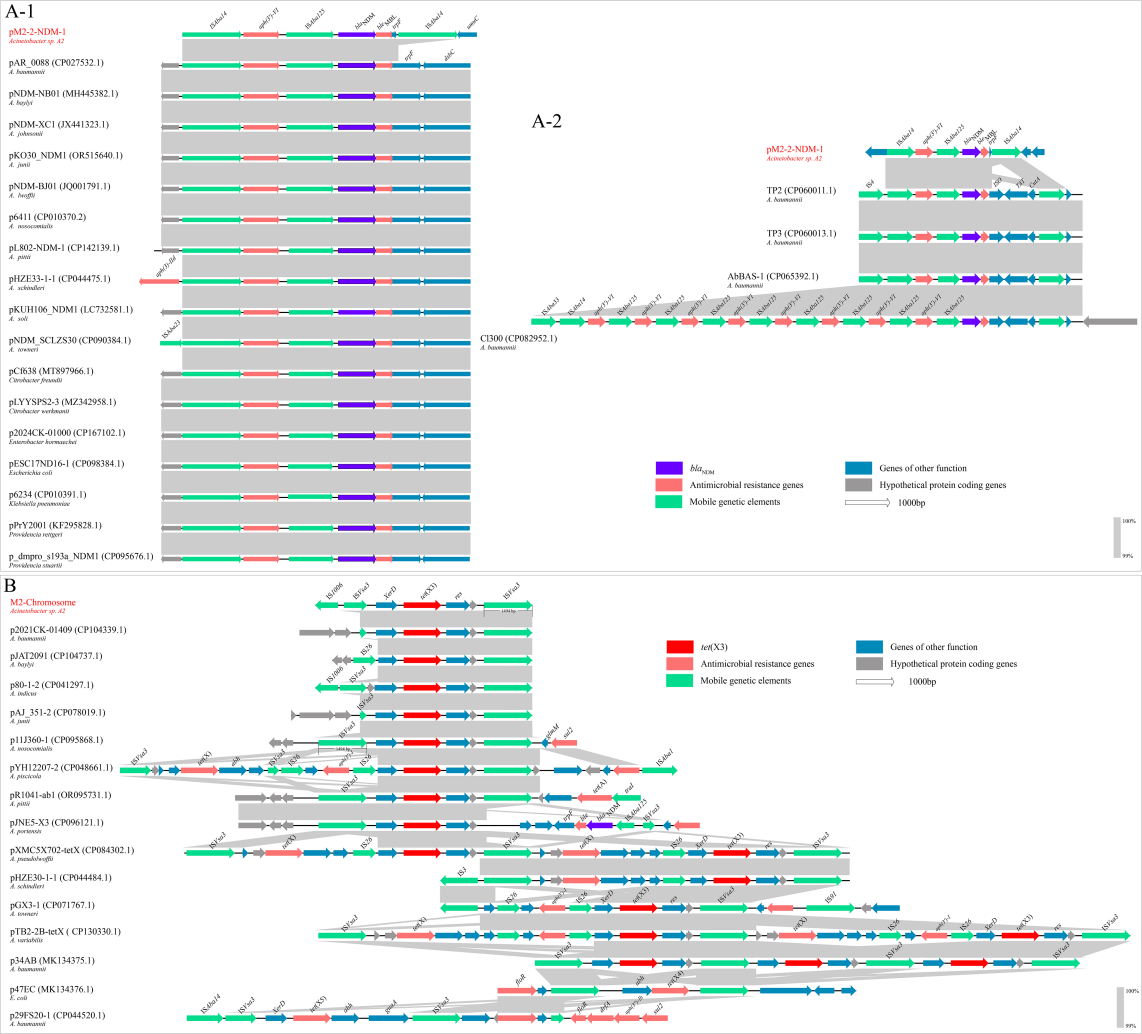
Comparison analysis. **(A-1)**: The comparison analysis between the rolling-circle-like structure and the similar segments of plasmids from the NCBI database. **(A-2)**: The comparison analysis between the rolling-circle-like structure and Tn*7382*. **(B)**: The comparison analysis of the *tet*(X3) skeleton.

The *bla*_NDM-1_ framework consisted of IS*Aba125*-*bla*_NDM-1_-*ble*_MBL_-*trpF* with a size of 2426 bp, located in the pM2-2-NDM-1 (**Figure 1**). We found that an *aph-(3’)-VI* was located upstream of *bla*_NDM-1_ framework. Two intact IS*Aba14* (1282 bp, orienting the same direction) flanking the structure of *aph-(3’)-VI*-IS*Aba125*-*bla*_NDM-1_-*ble*_MBL_-*trpF* formed a rolling-circle-like structure of 5966 bp, IS*Aba14*-*aph-(3’)-VI*-IS*Aba125*-*bla*_NDM-1_-*ble*_MBL_-*trpF*-IS*Aba14* (**Figure 1**). ISFinder analysis revealed that this IS*Aba14* (the group IS*150* of family IS*3*) had 99% identity with the reference sequence (1280/1282 bp) from *A. baumannii*. The BLASTn analysis of this rolling-circle-like structure in the NCBI database found that the plasmids of multiple bacterial species were high similar (>99% identity and coverage), including *Acinetobacter* genus (*A. baumannii*, *A. nosocomialis*, *A. baylyi*, *A. johnsonii*, *A. junnii*, *A. lwoffii*, *A. pittii*, *A. schindleri*, *A. soli*, and *A. towneri*), *Citrobacter* genus (*Citrobacter freundii* and *Citrobacter werkmanii*), *Providencia* genus (*Providencia rettgeri* and *Providencia stuartii*), *E. coli*, *Enterobacter hormaechei*, and *Klebsiella pneumoniae*. These plasmids (one plasmid genome selected from each bacterial species) and the pM2-2-NDM-1 were used to construct a comparison analysis (**Figure 5A-1**). Meanwhile, ISFinder was used to search downstream of the *bla*_NDM_ skeleton. The analysis found that IS*Aba14* frequently emerged upstream of the *bla*_NDM_ skeleton in the plasmids from the public database but rarely downstream of the *bla*_NDM_ skeleton. Furthermore, the BLASTn analysis on the UniProt database found that an intact *trpF* consisted of 639 bp, which could be identified in the plasmids from the public database. However, the *trpF* in the *bla*_NDM_ framework of pM2-2-NDM-1 was truncated by ISA*ba14* with a size of 114 bp. Tn*7382* is derived from Tn*125* and encompasses seven open reading frames (*aph-(3’)-VI*, IS*Aba125*, *bla*_NDM-1_ *, ble, iso, TAT, cutA*) enclosed by two direct copies of IS*Aba14* (Hamed et al., 2022). It was found in multiple chromosomes (TP2, TP3, AbBAS-1, and CI300) from *A. baumannii* and exhibited structural similarity to the rolling-circle-like structure in pM2-2-NDM-1. Thus, a linear comparison was built (**Figure 5A-2**). The analysis found three genes (*iso, TAT, cutA*) between *ble* and downstream IS*Aba14* in Tn*7382*. However, only one gene (fragmentary *trpF*) was located in the same site of pM2-2-NDM-1. Furthermore, an IS*4* or IS*Aba33* was located upstream of the left-hand IS*Aba14*. A similar location was not observed in pM2-2-NDM-1.

### The genetic context of *tet*(X3)

The *tet*(X3) framework, IS*Vsa3*-*XerD*-*tet*(X3)-*res*-IS*Vsa3*, with a size of 5819 bp, was located in the chromosome of strain M2. Two IS*Vsa3* flanked the structure of *XerD*-*tet*(X3)-*res*. The upstream IS*Vsa3* was a fragmentary element of 715 bp, and the downstream IS*Vsa3* was an intact element of 1494 bp. ISFinder analysis revealed that IS*Vsa3*, belonging to the IS*CR* family, originated from *Vibrio salmonicida*. The BLASTn analysis of this *tet*(X3) framework in the NCBI database found that the similar frameworks were mainly identified in the *Acinetobacter* genus and *E. coli*. The plasmid genomes with >99% identity and coverage to this framework were selected to construct the alignment analysis (**Figure 5B**). The analysis found that in addition to *tet*(X3), the IS*Vsa3* also mediated the formation of *tet*(X4) and *tet*(X5) frameworks. Most IS*Vsa3*-mediated *tet*(X3) frameworks were sourced from the *Acinetobacter* genus. An intact IS*Vsa3* was commonly located downstream of *tet*(X). However, either a fragmentary or intact IS*Vsa3* was located upstream of *tet*(X). In addition to IS*Vsa3*, an IS*26* frequently appeared upstream of *tet*(X). An IS element, IS*1006*, that is rarely reported to be associated with the *tet*(X) family emerged upstream of the left-hand IS*Vsa3*. Furthermore, IS*Vsa3* mediated the rolling-circle-like structures carrying *tet*(X3) (p34AB) or *tet*(X4) (p47EC).

## Discussion

Most *Acinetobacter* plasmids are considered non-conjugative due to the rarely observed transfer of plasmids. Although this study did not observe the horizontal transfer of plasmid carrying *bla*_NDM_ (pM2-2-NDM-1), it was predicted as conjugative. This may be attributed to laboratory systems omitting key microbial ecologic drivers, such as nutrient gradients, multispecies competitive dynamics, stress-induced epigenetic regulatory pathways, etc. These drivers are hard to replicate in the laboratory. This may be why the horizontal transfer of *Acinetobacter* plasmids is rarely observed. In addition to plasmid conjugation, the MGEs related to horizontal transfer of ARGs, including ICEs, p*dif* modules, and a rolling-circle-like structure, were identified in the pM2-2-NDM-1. A putative ICE region containing *oriT*, T4SS, T4CP, relaxase, integrase, and ARGs was identified. The genes ICEs carry common encoding antibiotic resistance determinants and virulence factors (Bellanger et al., 2014; Delavat et al., 2017) can confer the host with selective advantages, suggesting that ICE is a vital element for bacterial adaptation and evolution (Burrus et al., 2002; Bellanger et al., 2014; Wang et al., 2024). In ICEs, the T4SS and T4CP serve distinct yet complementary core functions (Bellanger et al., 2014; Delavat et al., 2017). The T4SS mediates the assembly of transport channels, and the T4CP orchestrates adaptor-mediated DNA delivery (Bellanger et al., 2014; Delavat et al., 2017). Their synergistic interaction critically governs the efficiency and specificity of conjugative transfer (Bi et al., 2012; Bellanger et al., 2014; Delavat et al., 2017; Wang et al., 2024). Typically, these elements carrying an integrase gene, a relaxase gene, and T4SS gene clusters are considered ICEs (Bi et al., 2012; Wang et al., 2024). The putative ICE region in pM2-2-NDM-1 carries the above elements. Thus, we consider that this ICE region is a fully functional ICE with the potential to transfer ARGs (*bla*_NDM-1_, *aac(3)-IId*, *aph(3’)-Ia*, and *tet*(39)) horizontally. Furthermore, six p*dif* sites and associated p*dif*-ARG modules have been detected in the pM2-2-NDM-1. The widespread distribution of the p*dif*-ARG module copes in the plasmid genomes of the *Acinetobacter* genus (Blackwell and Hall, 2017; Mindlin et al., 2019; Lin et al., 2020; Shao et al., 2023) indicates that p*dif*-ARG modules are associated with the horizontal transfer of resistance genes. In this study, the copies of p*dif*-*aac(3)-IId*-*aph(3’)-Ia* and p*dif*-*tet*(39) modules were identified in the plasmid genomes of the public database, suggesting the horizontal mobilization of these modules. The rare structure of the p*dif*-*msr*(E)-*mph*(E) module in the pM2-2-NDM-1 suggests that p*dif* modules are variable. This module carrying the *higA*-*higB* gene pair is critical for bacterial survival, stress adaptation, and pathogenicity. The common role of all types of p*dif* modules is to increase the fitness of their respective bacterial hosts in their habitats (Mindlin et al., 2019).

The *bla*_NDM_-positive strains were first retrospectively identified in 2005 from *A. baumannii* in an Indian hospital (Toleman and Walsh, 2012). In early isolates, *bla*_NDM_ is located within the intact Tn*125* transposon, leading to the hypothesis that Tn*125* is the ancestral transposon for *bla*_NDM_ *(* Kikuchi et al., 2022). The upstream region of Tn*125* carries ISs from families such as IS*5*/IS*30*, and frequent recombination of these IS elements has generated diverse genetic backgrounds (Toleman and Walsh, 2012; Acman et al., 2022). Studies (Kikuchi et al., 2022) found that multiple MGEs played critical roles in *bla*_NDM_ dissemination, including IS*3000*, IS*26*, IS*5*, IS*CR1*, Tn*3*, Tn*125*, and Tn*3000*. IS*Aba125*, a member of the IS*30* family, is typically located upstream of *bla*_NDM_ and forms a structure, IS*Aba125*-*bla*_NDM_-*ble*_MBL_-*trpF*-*dsbC*. It has been widely accepted that *bla*_NDM-1_ is regulated by a hybrid promoter containing the sequence from *bla*_NDM-1_ and IS*Aba125* (Kikuchi et al., 2022), and IS*Aba125* is commonly present in some form within *bla*_NDM_-positive isolates (Acman et al., 2022). In this study, two intact IS*Aba14* flank the *bla*_NDM_ skeleton to form a rolling-circle-like structure. Similar segments can be found in the plasmids of multiple bacterial genera, particularly in the *Acinetobacter* genus. However, these segments cannot be confirmed as rolling-circle-like structures due to the absence of downstream IS*Aba14*. The discrepancy between the rolling-circle-like structure and Tn*7382* focuses on the downstream region of *ble* in the *bla*_NDM_ skeleton, suggesting that this region is variable and unassociated with the *bla*_NDM_ expression. However, the upstream region of *ble* keeps a high similarity, suggesting that this region is potentially associated with the effective expression of *aph(3’)-VI* and *bla*_NDM_. The abundance of IS*Aba14* is present in the *Acinetobacter* genus (Hamed et al., 2022), and the Tn*7382* shows structural similarity to the rolling-circle-like structure mediated by IS*Aba14*, hinting that this rolling-circle-like structure has the potential for transmission.

IS*Vsa3* plays a pivotal role in the formation of the *tet*(X) framework, such as *tet*(X3), *tet*(X4), and *tet*(X5). Typically, full-length IS*Vsa3* is positioned downstream of *tet*(X), while the upstream region may observe intact/truncated IS*Vsa3* or other ISs. IS*Vsa3* participates in forming rolling-circle replication structures to mediate the horizontal mobilization of *tet*(X3) or *tet*(X4) *(* He et al., 2019). IS*Vsa3* also mediated *tet*(X) skeleton and other ARGs, such as *aph*, to form composite transposon. In addition to the *tet*(X) family, IS*Vsa3* is associated with multiple ARGs, such as *floR*, *tet(A)*, *aph(6)-Id*, *aph(3″)-Ib*, and *sul2 (* Lewis et al., 2023). Thus, monitoring IS*Vsa3* is critical for understanding the distribution and spread of ARGs, particularly *tet*(X). Additionally, we found that an IS element, IS*1006*, that is rarely seen in *the tet*(X) skeleton closely links with IS*Vsa3.* This link was also observed in the p80-1–2 of *A. indicus* (**Figure 5B**). In this work, the strain M2 was susceptible to tetracycline antibiotics (doxycycline, tigecycline, and omadacycline). The BLASTn analysis between the *tet*(X3) sequence of strain M2 and the reference sequence (NG_048307, 1361 bp) from the NCBI nucleotide database found that these sequences were identical (100% identity and coverage). This silent phenotype was also observed in most *Acinetobacter* isolates carrying *tet*(X) (including *tet*(X3) and *tet*(X5)) in our laboratory. These strains have a common characteristic that a fragmented IS*Vsa3* or an IS element previously unreported to associate with the *tet*(X) family, IS*1008*, was located upstream of the *tet*(X) skeleton. This change in structure is attributed to frequent recombination events upstream of *tet*(X), which may lead to the downregulation of the expression level of *tet*(X3). The expression of the resistance gene is regulated by a hybrid promoter, which has been observed in *bla*_NDM_ *(* Kikuchi et al., 2022). The subsequent evolutionary trajectory may fix the gap. Furthermore, in light of strict antibiotic restrictions in bovine production, plasmids may downregulate *tet*(X) expression to reduce fitness costs and facilitate host survival until environmental triggers activate resistance mechanisms. This strategy conforms to the “stealth-to-threat” model of plasmid evolution, in which genetic cargo is closed until environmental pressure demands its activation.

Livestock is recognized as a critical reservoir for carbapenem-resistant bacteria (CRB) (Wang et al., 2017), yet investigations into bovine production remain limited. This is the first report of the co-occurrence of *bla*_NDM-1_ and *tet*(X3) genes in a strain belonging to a novel species of the *Acinetobacter* genus from bovine production. This co-occurrence reflected the functional convergence of carbapenem and tetracycline resistance mechanisms. This convergence may be caused by dual selection pressures mediated by antimicrobial usage patterns. Although carbapenems and tigecycline undergo strict usage controls, the extensive application of tetracyclines and β-lactams in bovine production leads to the co-occurrence of *bla*_NDM_ and *tet*(X), such as amoxicillin and tetracycline as the major veterinary antibiotics (Ma et al., 2024). Thus, we consider that the frequently observed co-occurrence of these genes represents an established dissemination network rather than sporadic acquisition events. This co-occurrence may be more frequently observed in the future. Furthermore, the co-occurrence of *bla*_NDM_ and *tet*(X) in a novel species of the *Acinetobacter* genus should cause public concern because this co-occurrence highlights the presence of substantial undiscovered co-occurrence of high-risk ARGs in the agroecological system. This will inevitably lead to an elevated risk of disseminating high-risk ARGs into human populations.

## Methods

### Sampling and microbial identification

The sludge was collected from a commercial beef cattle farm in Hebei province, China. The strain was isolated from MacConkey (Huan Kai Microbial, China) agar plates supplemented with 2 μg/mL meropenem and cultured at 37°C for 16 hours. PCR amplification and Sanger sequencing verification were performed for *bla*_NDM_ and *tet*(X) genes (Wang et al., 2017; He et al., 2019).

### Whole-genome sequencing

The long-read sequencing was executed on the Oxford Nanopore platform. Briefly, the genome was sequenced using Oxford Nanopore and the DNBSEQ platform. The resulting corrected reads were carried out by hybrid assembly in combination with DNBSEQ short reads. The assembled genome was checked for completeness and contamination using the CheckM (v1.2.3) of the NCBI annotation service.

### Identification of bacterial species

The identification of bacterial genus was performed by MALDI-TOF-MS and 16s rRNA (Kim et al., 2010; Singhal et al., 2015). The identification of bacterial species was executed on the NCBI database using ANI match of the NCBI annotation service, on the DSMZ platform (Leibniz Institute DSMZ: Welcome to the Leibniz Institute DSMZ) using the type strain genome server (TYGS), and on the JSpeciesWS platform (JSpeciesWS - Taxonomic Thresholds) using tetra correlation search (TCS). Furthermore, we used the mash match tool to identify bacterial species on the Pathogenwatch platform (Pathogenwatch | A Global Platform for Genomic Surveillance) (Argimón et al., 2021). The ANI calculation was executed on the EZBioCloud platform (ANI Calculator | Ezbiocloud.net) using the OrthoANIu algorithm (Yoon et al., 2017). The GGDC platform (ggdc.dsmz.de/ggdc.php#) was used to calculate the dDDH value.

### Antimicrobial susceptibility testing

The minimum inhibitory concentration (MIC) of 20 antibiotics (meropenem, aztreonam, ampicillin, ceftazidime, cefepime, gentamicin, chloramphenicol, colistin, kanamycin, fosfomycin, ciprofloxacin, sulfamethoxazole, azithromycin, tetracycline, doxycycline, tigecycline, imipenem, ertapenem, amikacin and omadacycline) was performed using microdilution of Mueller-Hinton broth (Huan Kai Microbial, China). The testing concentration range is 2 mg/L to 1024 mg/L. Results were determined according to Clinical and Laboratory Standards Institute (CLSI) documents M100-S34 (2024). Since there is no established breakpoint for tigecycline and omadacycline resistance in *Acinetobacter* spp., their breakpoint was determined by FDA-defined interpretive criteria for *Enterobacteriaceae (*R≥ 8 μg/mL for tigecycline and R≥ 16 μg/mL for omadacycline*).*

### Conjugation assay

Conjugation assays were conducted based on the filter mating method using *E. coli* J53 (sodium azide-resistant) and *Salmonella* LGJ2 (rifampicin-resistant) as recipients. A donor-recipient mixture of 10:1 (the ratio 10:1 of the donor (M2) to the recipient (J53 or LGJ2)) was incubated on a 0.22-μm filter membrane at 35°C for 16h, then moved to MacConkey plates supplemented with donor resistance (0.5 mg/L meropenem) and recipient resistance (100 mg/L sodium azide or 200 mg/L rifampicin). The ratio 5:1 and 15:1 of the donor to the recipient were supplementarily tested. At least three attempts were made for each parameter, and three parallel experiments were made for each attempt.

### Phylogenetic construction

Core genomes were extracted using Roary (GitHub - sanger-pathogens/Roary: Rapid large-scale prokaryote pan genome analysis). Recombination was filtered using Gubbins (GitHub - nickjcroucher/gubbins: Rapid phylogenetic analysis of large samples of recombinant bacterial whole genome sequences using Gubbins). Filtered polymorphic sites were employed to build a tree on the PhyML platform (GitHub - stephaneguindon/phyml: PhyML – Phylogenetic estimation using (Maximum) Likelihood). The iTOL (iTOL: Interactive Tree Of Life) visualized this tree with the corresponding features of each genome.

### Bioinformatics analysis

ResFinder (v4.6.0) was used to screen all known acquired AMRs (Bortolaia et al., 2020). The threshold of ARG identification was set to 90%, and the minimum length was set to 80%. Plasmid typing was carried out on the Pathogenwatch platform using homology-based alignment and on the Galaxy (Galaxy | China) platform using MOB-typer. The PLSDB database (PLSDB) was used to analyze the plasmid characterization further. The VFDB was used to search for the genes encoding VFs (VFDB: Virulence Factors of Bacterial Pathogens) (Liu et al., 2022). Genome annotation was performed using the RAST genome annotation service (RAST Server - RAST Annotation Server) (Overbeek et al., 2014), with manual refinement using ORFfinder (ORFfinder Home-NCBI), UniPort (UniProt), and ISFinder (ISfinder) (Siguier et al., 2006). The p*dif* module was identified using PdifFinder (Home) (Shao et al., 2023). ICEfinder (ICEfinder) was employed to detect the *oriT*, integrase, relaxase, T4CP, T4SS, and the other associated components (28). The circular and linear comparisons were created using BRIG (ver. 0.95) and Easyfig (ver. 2.2.5).

## Data Availability

Genome assemblies of the strain M2 have been deposited in the NCBI database under BioProject accession no. PRJNA1228993.
